# Phylogenetic evidence for nationwide expansion *Brucella melitensis* lineages drives the re-emerging and epidemic of human brucellosis in Jiangsu, China

**DOI:** 10.3389/fcimb.2025.1603234

**Published:** 2025-09-02

**Authors:** Weixiang Wang, Zhou Lu, Ge Teng, Zikang Yan, Lan Huang, Zhongming Tan, Zhiguo Liu, Songning Ding, Zhenjun Li

**Affiliations:** ^1^ Infectious Disease Prevention and Control Department, Nanjing Municipal Center for Disease Control and Prevention, Nanjing, Jiangsu, China; ^2^ National Health Commission (NHC) Key Laboratory of Enteric Pathogenic Microbiology, Jiangsu Provincial Center for Disease Control and Prevention, Nanjing, Jiangsu, China; ^3^ Microbiology Laboratory, Nanjing Municipal Center for Disease Control and Prevention, Nanjing, Jiangsu, China; ^4^ School of Public Health, Southeast University, Nanjing, Jiangsu, China; ^5^ National Key Laboratory of Intelligent Tracking and Forecasting for Infectious Diseases, National Institute for Communicable Disease Control and Prevention, Chinese Center for Disease Control and Prevention, Beijing, China

**Keywords:** human brucellosis, *Brucella melitensis*, WGS-SNP, phylogeny analysis, cgMLST

## Abstract

**Objective:**

Human brucellosis has re-emerged as a major public health threat in Jiangsu Province, but the sources and transmission dynamics of circulating strains remain poorly understood.

**Methods:**

In this study, we integrated conventional biotyping, whole-genome sequencing single-nucleotide polymorphism (WGS-SNP) analysis, and core genome multilocus sequence typing (cgMLST) to investigate the phylogenetic relationships of *Brucella melitensis* in the region.

**Results:**

Among 89 isolates analyzed, all were confirmed as *B. melitensis* (16 as biovar 1 and 73 as biovar 3), with a widespread geographic distribution across 15 cities in Jiangsu and adjacent areas, indicating extensive regional dissemination. All strains belonged to sequence type 8 (ST8) and genotype group II, clustering within the East Mediterranean lineage. Genomic resolution classified these strains into five SNP clades (C-I to C-V) and 17 SNP-based genotypes (STs), revealing a ladder-like phylogenetic structure. The lack of distinct geographic clustering suggests frequent cross-regional transmission, likely facilitated by the movement of infected sheep and goats. Phylogenomic analysis through cgMLST revealed distinct clustering of the 17 STs into two major groups (G-I and G-II), with 15 STs (88.2%) showing high genetic concordance between Jiangsu isolates and strains from China’s northeastern and northwestern. This compelling genomic evidence establishes that the current human brucellosis epidemic in Jiangsu is being driven by the nationwide expansion of dominant *B. melitensis* lineages.

**Conclusion:**

The findings provide crucial insights into the infection sources and interregional transmission dynamics of brucellosis in southern China, highlighting the significant role of domestic animal movement in pathogen dissemination, demanding coordinated cross-regional interventions including strict implementing intervention strategies and enhance disease surveillance.

## Introduction

Brucellosis is a common zoonotic disease caused by *Brucella* spp., which are Gram-negative, non-motile, non-spore-forming, slow-growing, and facultative intracellular bacteria (Di Bonaventura et al., 2021). Brucellosis, also known as undulant fever or Malta fever, was first reported in the 1850s in Malta from a patient who was said to have acquired the infection through consumption of infected goat’s milk (Hull and Schumaker, 2018). In 1886, David Bruce first isolated and identified *Brucella melitensis* from the spleens of four soldiers as the causative agent of the disease (Aleixo et al., 1999). Currently, 12 *Brucella* species have been identified, including six classical species and six novel species (Bricker et al., 2000). Among, four strains exhibit pathogenicity in humans and animals, *B. melitensis* is the most infectious, the other strains are *Brucella suis* and *Brucella abortus* and more recently human cases being infected with *Brucella cetaceae* have been reported (Gorvel, 2008; Orsini et al., 2022). Direct or indirect contact with infected animals and consumption of unpasteurized dairy products are the main transmission routes of brucellosis to humans, and people may also be infected by inhalation of contaminated dust or aerosols (Al Dahouk et al., 2003; Khalafalla, 2023). Domestic animals and wildlife, including goats, sheep, cattle, deer, and camels, are natural hosts of *Brucella* spp (Liu et al., 2020). The persistent worldwide prevalence of human brucellosis has caused serious public health concerns and vast economic losses in the farming industry (Singh et al., 2018).

Human brucellosis is prevalent worldwide, and an evidence-based report suggests that the global incidence of human brucellosis is 2.1 million new cases every year, with the highest incidence of human brucellosis in the Middle East and Central Asia (Laine et al., 2023). Furthermore, human brucellosis has become a serious public health challenge, and the cases reported in all 31 provinces in mainland China and the affected regions have expanded from northern to southern, including Jiangsu province (Lai et al., 2017). During the years 2006-2021, 1,347 brucellosis cases were reported in Jiangsu Province, with an average annual incidence of 0.1036 per 100,000 individuals (Zhang et al., 2023). Four cluster cases of brucellosis from one family were reported in Shuyang County, Jiangsu Province, China, and isolates from the four patients were indistinguishable by MLVA profiling, displaying a unique type in Jiangsu Province (Tan et al., 2015). Whole-genome sequencing single-nucleotide polymorphism (WGS-SNP) analysis is a widely used genotyping tool for identifying the transmission pattern and phylogenetic inferences of *Brucella* strains (Janowicz et al., 2018; Xue et al., 2023). And, cgMLST scheme that is applicable in *Brucella* molecular epidemiology and helps in accurately tracking and thus controlling the sources of infection (Abdel-Glil et al., 2022). In this study, we performed WGS-SNP and cgMLST analyses on *Brucella melitensis* strains isolated from human patients in Jiangsu Province to elucidate their transmission patterns and phylogenetic relationships, thereby providing critical insights for developing targeted surveillance and control strategies.

## Materials and methods

### *Brucella* strain isolation and convention identification

A total of 89 *Brucella* spp. was isolated from blood samples collected from patients with suspected brucellosis in Nanjing City. *Brucella* strains were isolated according to a standard bacteriology protocol (Yagupsky et al., 2019). Briefly, five (~10) fresh blood samples were collected from suspected patients, injected into a diphasic medium in a biosafety cabinet, and then incubated in a fully automated blood culture system (BACTECTM9000, BD) for at least two weeks. The culture-positive samples were screened by real-time PCR (Al Dahouk et al., 2007), transferred to new medium for purification and isolation, and subjected to AMOS-PCR and agglutination with anti-A and M monospecific sera tests to determine the species/biovars (Eltigani et al., 1991; Zhou et al., 2021).

### DNA prepare, whole-genome sequencing and pangenome analysis

DNA preparation, quality assessment, and Whole-Genome Sequencing were based on a previous study (Xue et al., 2023). Genomic DNA was prepared from heat-inactivated (80°C, 10 min) pure cultures using the Fast Pure Bacterial DNA Isolation Mini Kit [Nanjing Novozymes Biotechnology Co., Ltd. (Nanjing, China)] according to the manufacturer’s instructions. After DNA preparation, all DNA samples were fragmented by sonication to a size of 350bp, sequencing library preparation was performed using the Nextera XT library preparation kit (Illumina Inc., San Diego, CA, USA), and whole-genome sequencing of the DNA sample was performed on the MGISEQ-2000 platform. Briefly, raw data were filtered and assembled using the CLC Genomics Workbench V23.0.1 software (QIAGEN, Hilden, Germany). A summary table of genome statistics (size, GC content, N50, contig counts, and gene counts) is provided in **Supplementary Table S1**, highlighting assembly quality and uniformity among isolates. Based on the Virulence Factor Database (VFDB) sequences with >98% coverage and identity, Virulence-associated genes were identified. Genomic mobile elements including IS711-family insertion sequences, plasmids, and integrative conjugative elements (ICEs) were comprehensively analyzed to evaluate horizontal gene transfer capacity. The IS elements were annotated using ISfinder (e-value cutoff 1e-5), plasmid contigs were identified through alignment with PlasmidFinder (95% identity threshold), and ICEs were predicted using ICEberg 2.0 with manual curation of conserved features (integrases, conjugation modules). Antimicrobial resistance (AMR) genes were systematically screened across all isolates using a AMRFinderPlus (v3.10.30) with default parameters. Only hits meeting ≥98% sequence identity and ≥98% coverage thresholds were considered valid. Panaroo tools (Tonkin-Hill et al., 2020) was used to identify core, and unique genes across the isolates, and interpreted their functional relevance.

### Genome epidemiology investigation and phylogeny analysis of *B. melitensis* strains

Genome epidemiology investigation and phylogeny analysis of 89 *Brucella melitensis* strains was performed based on the Microbial Genomics Module in the CLC Genomics Workbench V23.0.1 software (QIAGEN, Hilden, Germany), including MLST and WGS-SNP analysis. Briefly, raw sequence reads were imported into CLC Genomics Workbench (version 22.0). Quality trimming was performed using the built-in Trim Sequences tool with fault parameters. Processed reads were aligned to the *B. melitensis* 16M reference genome (GCA_000007125.1) using Map Reads to Reference tool (default parameters). Duplicate reads were removed using the Remove Duplicate Mapped Reads tool (Strict Mode). High-confidence SNPs were identified using the Basic Variant Detection tool, and a core-genome SNP alignment was generated by extracting all high-quality SNPs and concatenating them into a single FASTA file (Create Alignment from the Variants tool). Sites with missing data (>10% isolates) or low confidence were removed, and the final alignment was exported in the PHYLIP format. Maximum likelihood (ML) phylogeny was inferred using the RAxML plugin (v8.2.12) integrated into the CLC with the GTR+G (selected via ModelTest based on BIC) substitution model. Maximum-likelihood trees of the 89 *B. melitensis* strains were constructed using a maximum-likelihood phylogenetic algorithm with 1,000 bootstrap replicates (Minh et al., 2020). As described previously (Janowicz et al., 2018), a global scale core-genome MLST (cgMLST) approach was employed to construct a minimum spanning tree, revealing the regional transmission patterns. The resulting Newick tree file was imported into the iTOL v6.5.7 for visualization (Letunic and Bork, 2021).

## Results

### The species/biovars and distribution of 89 *Brucella* strains

**Figure 1:**
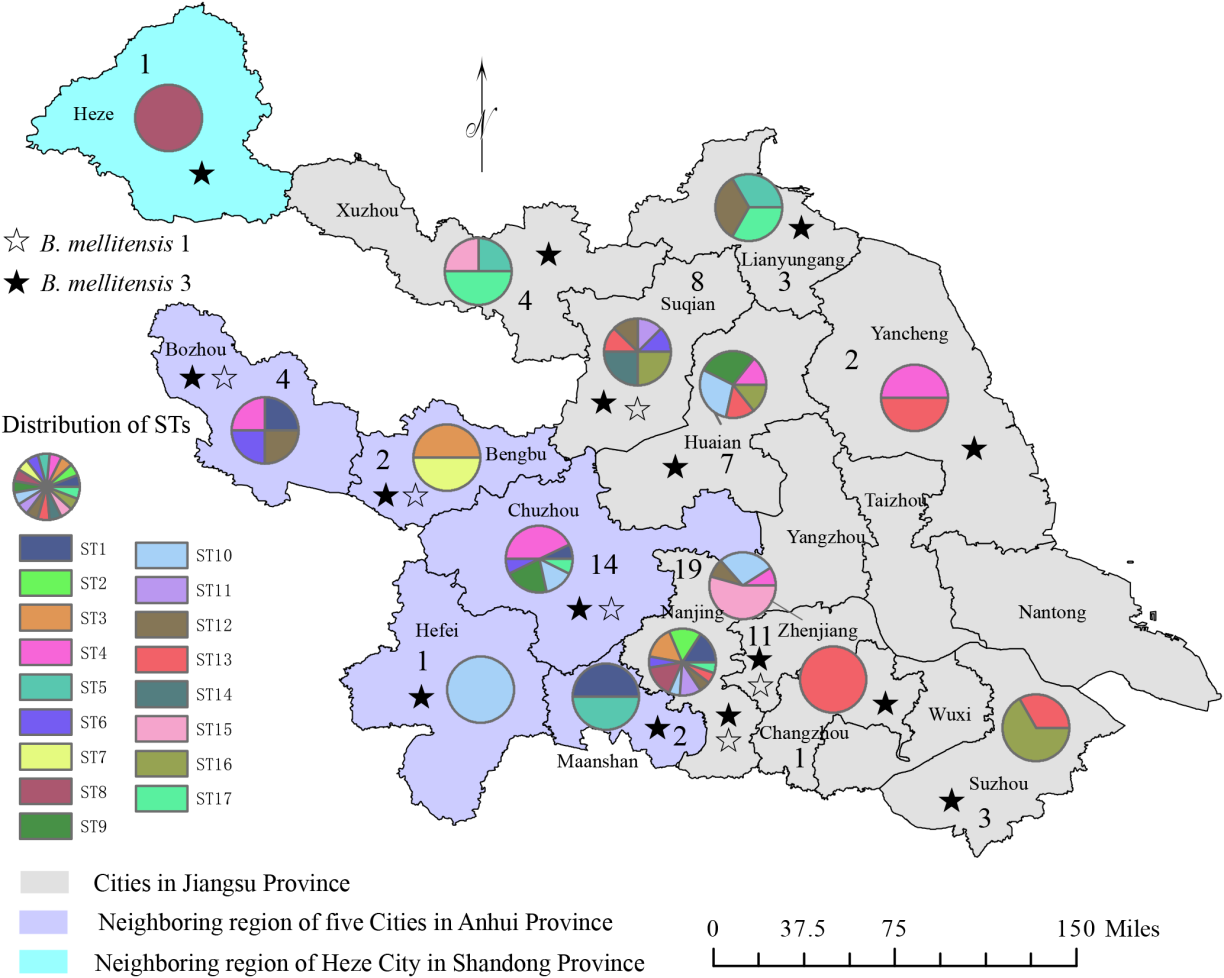
The distribution pattern of the number of strains, species/biovars, and STs of 89 *B*. *melitensis* from Jiangsu Province. The figure annotations are as follows: numerical values represent strain counts, ☆ symbols denote *B*. *mellitensis* bv. 1 strain, ★ symbols indicate *B*. *mellitensis* bv. 3 strains, and the color-coded pie chart illustrates the distribution of the 17 STs.

Based on AMOS-PCR amplification, a special 731 bp band was observed in all samples, implying that the strains were all *Brucella melitensis* strains. Furthermore, agglutination with anti-A and M monospecific sera tests showed that 16 strains of *B. melitensis* bv. 1 and 73 strains were identified as *B. melitensis* bv. 3 (**Figure 1**; **Table 1**). Based on the isolated location, 89 strains were isolated from three provinces and 15 cities (counties), one from Shandong, 23 from Anhui province, and 58 from Jiangsu provinces. The two provinces are adjacent to Naning, Jiangsu province, and patients were located near Nanjing for convenient medical treatment (**Figure 1**). These data indicate that *B. melitensis* bv. 3 was widely prevalent in Nanjing and bordering regions.

**Table 1:** The species/biovars, geographic distribution of 89 *B. melitensis* strains in this study.

Province	Location	Year	Number of strains	AMOS-PCR	A serum	M serum	Biovars
Jiangsu	Nanjing (4)	2018-2022	16	731bp	N	P	bv. 1
	Suqian (1)						
	Zhengjiang (2)						
Anhui	Chuzhou (5)						
	Bozhou (1)						
	Benbu (1)						
Unknown	Unknown (2)						
Anhui	Chuzhou (9)	2017-2022	73	731bp	P	P	bv. 3
	Bozhou (3)						
	Maanshan (2)						
	Hefei (1)						
	Benbu (1)						
Shandong	Heze (1)						
Jiangsu	Nanjing (15)						
	Zhengjiang (9)						
	Huaian (7)						
	Suqian (7)						
	Xuzhou (4)						
	Suzhou (3)						
	Liangyungang (3)						
	Yancheng (2)						
	Changzhou (1)						
Unknown	Unknown (5)						

P, positive; N, Negative.

### Genome summary, virulence associated genes analysis and pangenome of *B. melitensis*

**Figure 2:**
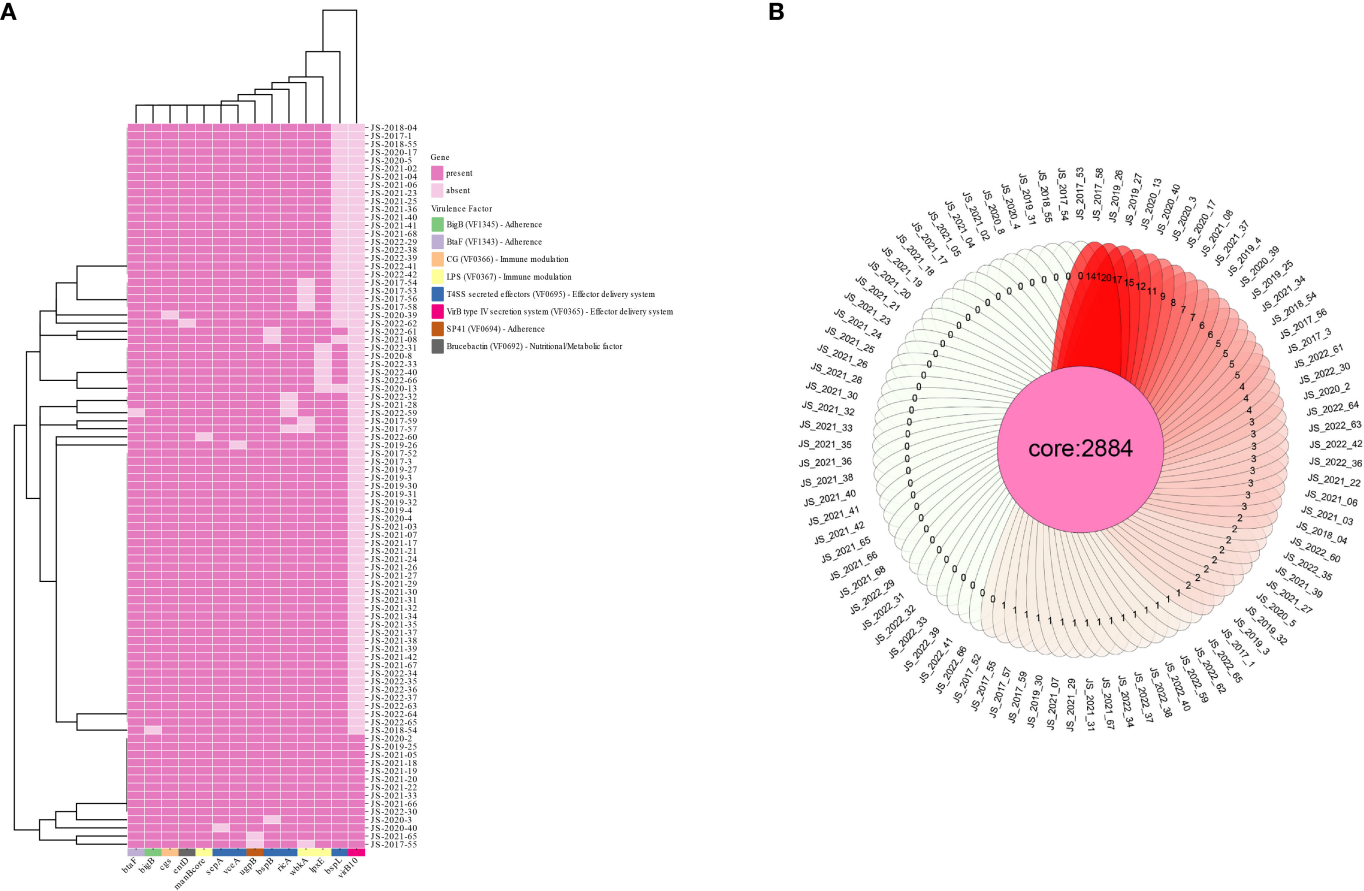
Distribution profile of virulence-associated genes **(A)** and core genes **(B)** among B. melitensis strains.

The analyzed strains demonstrated high genomic conservation with *B. melitensis* 16M, sharing an Average Nucleotide Identity (ANI) of 99.86–99.89%, confirming their close phylogenetic relationship. Genome assemblies showed moderate to high continuity, with scaffold counts ranging from 20 to 49, N50 values of 202,239–297,774 bp, and N70 values of 104,781–189,498 bp. All strains maintained a conserved genome size (~3.2 Mb; range: 3,238,490–3,389,613 bp) and encoded 1,412 protein-coding genes, consistent with the characteristic genomic architecture of *Brucella* spp. (**Supplementary Table S1**). Virulence-associated gene analysis revealed remarkable stability across the 89 strains, with 71–73 virulence genes retained per isolate. Notably, 14 genes exhibited partial absences, most frequently virB10 (missing in 84.3% of strains), followed by bspL (30.3%), wbkA (7.9%), and lpxE (6.7%) (**Figure 2A**). The remaining ten genes showed even lower absence frequencies (<5%). Functional annotation identified these variable genes as critical components of the VirB type IV secretion system (T4SS), T4SS-secreted effectors, and lipopolysaccharide (LPS) biosynthesis pathways—key determinants of bacterial virulence and host immune evasion. Antimicrobial resistance (AMR) screening detected only mprF (defensin resistance) across all strains, with no other AMR genes identified. Pangenome analysis delineated 2,884 core genes shared among all isolates, while strain-specific unique genes ranged from 1 to 141 (**Figure 2B**), reflecting both genomic stability and niche-specific adaptations.

### The epidemiology correlation analysis of 89 *Brucella* strains

**Figure 3:**
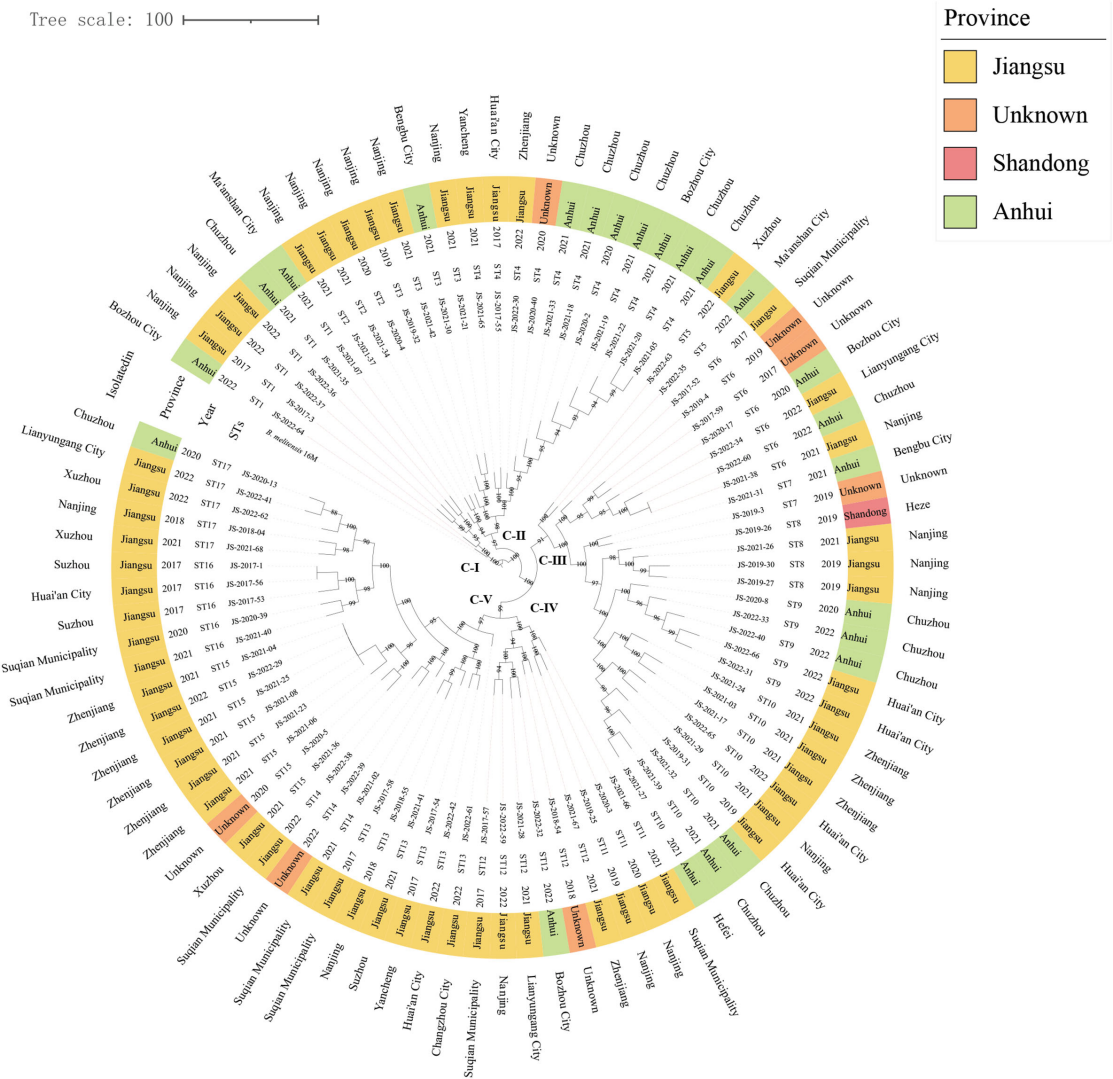
The maximum-likelihood tree generated by cgSNP matrix of 89 *B*. *melitensis* on county scale. The phylogeny trees of 89 *B. melitensis* strains were calculated using TreeBeST based on the Maximum-Likelihood Phylogenies (PHYML) algorithm with 1,000 bootstrap replicates. The maximum-likelihood tree depicts five major cgSNP-based clades (CI–CV), with bootstrap values (>85%) shown at branch nodes. The concentric circles (inner to outer) represent: Strain names, cgSNP genotypes (ST), Isolation year, Province (color-coded), and Geographic origin (prefecture/city).

Using both MLST-9 schemes, all strains were consistently classified as sequence type 8 (ST8). cgSNP analysis delineated the 89 *B. melitensis* into five phylogenetically distinct SNP clades (C-I to C-V) containing 17 SNP genotypes (STs), with C-I represented by ST1, C-II encompassing ST2-ST4, C-III comprising ST5-ST10 as the most diverse clade, C-IV containing ST11-ST12, and C-V including ST13-ST17. Notably, strains within each clade exhibited broad geographical distributions across multiple provinces, with the C-III clade demonstrating particularly extensive spread across Anhui, Shandong, and Jiangsu provinces. At finer spatial resolution, only ST2 showed restriction to a single region, while the remaining 16 STs contained strains from ≥2 regions collected across different time periods. The observed ladder-like phylogenetic topology and absence of geographical clustering strongly suggest recurrent cross-regional transmission events originating from multiple common infection sources, highlighting the complex dissemination patterns of *B. melitensis* in the study area (**Figure 3**).

### Global cgMLST phylogenetic analysis of *Brucella melitensis* strains

**Figure 4:**
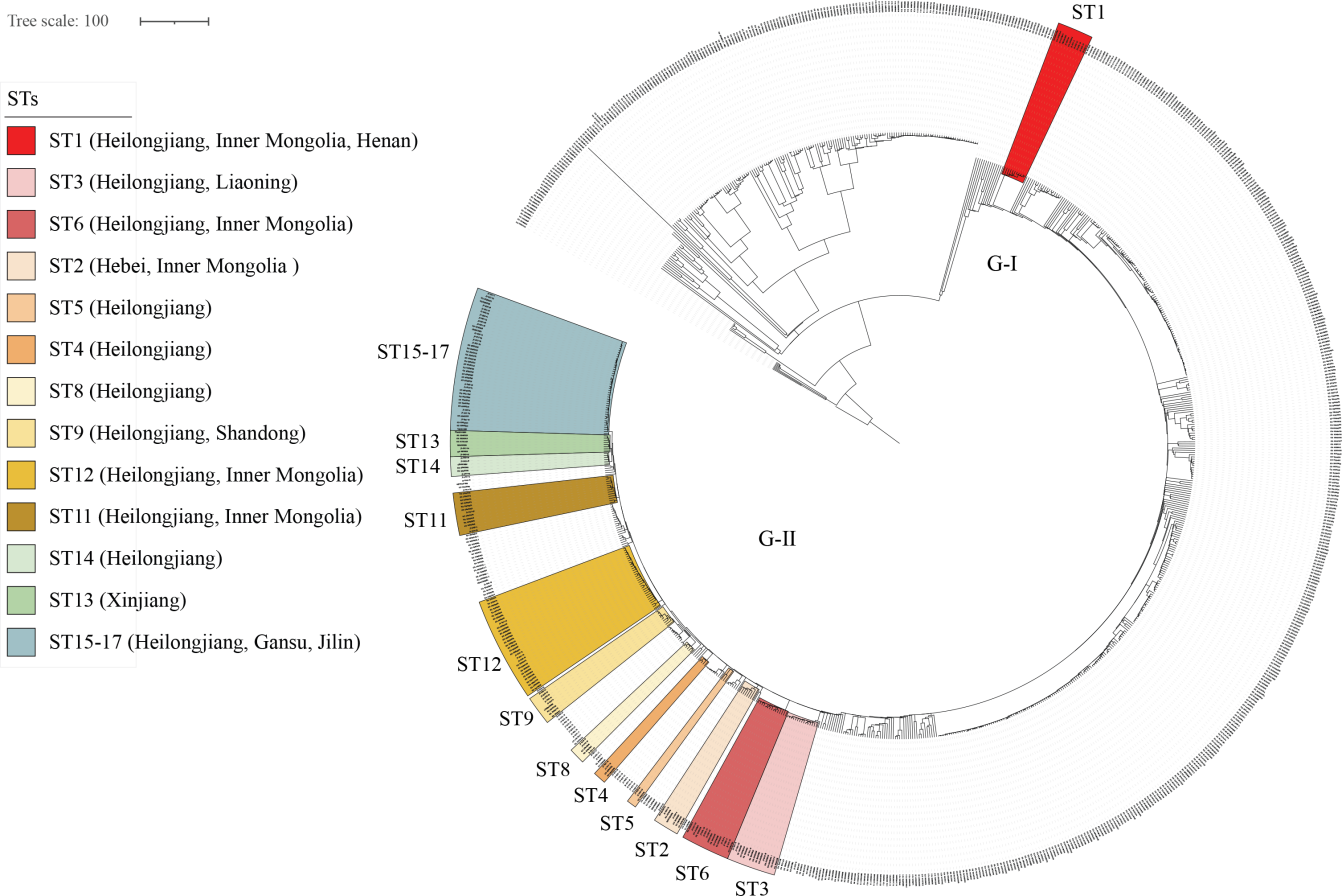
Global phylogenetic analysis of *Brucella melitensis* strains based on core genome multilocus sequence typing (cgMLST). Strains in each STs from study (highlighted) exhibit close phylogenetic relationships with isolates from the indicated regions (labeled by province in brackets), the tree topology reveals population structure and evolutionary divergence among global *B. melitensis* lineages, color-coded bars demonstrate complete cgSNP genotype matching between some or all strains of these sequence types (STs) and geographically distinct isolates from other provinces. The remaining unlabeled strains represent publicly available sequences sourced from GenBank.

Global cgMLST-based phylogenomic analysis demonstrated that all 89 strains consistently clustered within Genotype II of the East Mediterranean lineage, with the 17 STs segregating into two primary groups: ST1 and the remaining 16 STs. Strikingly, 15 of these STs exhibited genotype identities matching strains from multiple northern Chinese regions (**Figure 4**). Phylogenetic analysis revealed ST1’s close relatedness to Heilongjiang, Inner Mongolia, and Henan strains, while ST2-4 (C-II) formed a distinct clade with Heilongjiang, Liaoning, Hebei, and Inner Mongolia isolates; ST5-10 (C-III) showed tight clustering with Heilongjiang, Shandong, and Inner Mongolia strains; ST11-12 (C-IV) demonstrated high genetic concordance with Heilongjiang and Inner Mongolia isolates; and ST13-17 (C-V) exhibited clear phylogenetic linkages to strains from Heilongjiang, Xinjiang, Jilin, and Gansu (**Figure 4**). These results collectively indicate remarkable genetic homogeneity between the studied strains and northern Chinese (northeast/northwest) *Brucella* populations, strongly suggesting that the Nanjing-area human brucellosis epidemic likely originated through introduction of northern infection sources rather than representing an endemic focus.

## Discussion

Human brucellosis is a re-emerging disease in Jiangsu province. Sporadic cases were reported after 2006, and the number of reported cases has significantly increased since 2011 (Zhang et al., 2023). However, the species/biovars, origin, and transmission patterns of circulating *Brucella* strains remain unclear. Bacteriology, bio-typing, and WGS-SNP analyses were applied to uncover the original and transmission patterns of circulating *Brucella* strains. Our investigation showed that *B. meitensis* strains were prevalent in Jiangsu province and neighboring regions, including Anhui and Shandong provinces, dominated by *B. melitensis* bv. 3. Previous studies have shown that human brucellosis has changed significantly over the past decades, and *B. melitensis* strains have become a dominant circulating species in China, and it has expanded from north to south regions (Zhu et al., 2020). An outbreak caused by *B. melitensis* at Jinchi Biotechnological Company engaged in collecting and disposing of kitchen waste from catering units in Lianyungang has been reported (Zhang et al., 2020). Brucellosis has become an important public health issue in Anhui Province, serum and milk samples obtained from goats in different regions of Anhui province were tested, the investigation frequency of brucellosis using RBPT, SAT, MRT, and PCR methods was 3.9% (n=7), 4.45% (n=8), 11.67% (n=7), and 86.67% (n=156), respectively (Rahman et al., 2019). These data show that ruminant brucellosis has been a serious public health concern in Jiangsu and Anhui provinces.

MLST analysis showed that all strains belonged to ST8, which is the dominant ST in the northern region (Sun et al., 2016). ST8 is the prevalent sequence type and the transmission of osteoarthritis-associated *B. melitensis* among different geographical areas in Inner Mongolia (Zhu et al., 2024). In Guangxi province, the population structure of *Brucella* strains had changed considerably; ST17 and ST21, two previously predominant populations, appeared to have been replaced by the recently emerging ST8 group (Liu et al., 2019). A previous report demonstrated that the cause of an outbreak was the plentiful influx of unchecked sheep from the northern part of China, and the employees in the process of sheep slaughtering or trading lacked effective prevention programs (Xiang et al., 2014).

WGS-SNP analysis revealed that 89 strains were divided into five SNP clades and 17 SNP genotypes (STs); each clade formed a ladder topology, with strains lacking apparent territory ownership, implying that cross-region cases occurred due to common sources of infection. Whole-genome SNP analysis showed that four *B. melitensis* strains were closely related to strains from China’s northern provinces, and the source of infection was partly human brucellosis in this province, which may have been from these regions (Li et al., 2020). In Shaanxi, multiple cross-county brucellosis outbreak events are driven by multiple indigenous circulating *B. melitensis* lineages (An et al., 2024).

The global phylogeny analysis showed that strains displayed a high genetic homogeneity with strains from multiple northern provinces, implying that multiple *B. melitensis* strain lineages were imported due to animal trade that co-drove the human brucellosis epidemic in Jiangsu. According to their territorial affiliation between the five clade strains, the absence of a clear differentiation suggests that strains continuously expanded and spread in Jiangsu province and neighboring regions. Similarly, 110 Chinese animal *B. melitensis* strains were grouped into five clusters, reflecting the existence of multiple lineages, and Chinese lineages were more closely related to strains collected from East Mediterranean and Middle East countries, such as Turkey, Kuwait, and Iraq (Sun et al., 2017). The active transfer and trade of animals (sheep and goats) between these regions is a reasonable explanation. A relevant survey showed that this province mainly breeds and raises its own cattle and sheep, the imported sheep come from Inner Mongolia, Shandong, Anhui and other provinces (Xu and Wang, 2018). The lack of geographic clustering among Jiangsu strains provides strong evidence for frequent cross-regional livestock movements, which significantly compromises local disease control efforts. The observed high genetic concordance with northern Chinese strains particularly highlights the need for an integrated One Health approach to effectively manage transmission risks at the human-animal interface. Key interventions should include: (1) implementing mandatory testing and quarantine protocols for livestock imported from endemic regions, (2) establishing real-time genomic surveillance systems to accurately trace outbreak origins, and (3) enhancing farmer education programs to mitigate high-risk behaviors such as direct contact with infected animals and consumption of unpasteurized dairy products (Liu et al., 2023). Finally, to effectively curb the southward spread of brucellosis, it is essential to strengthen inter-sectoral collaboration between agriculture and public health authorities while enhancing inspection and quarantine measures for introduced livestock.

## Conclusion

At present, bacteriology and WGS-SNP have been applied to uncover the origin and transmission of *Brucella* strains, underscoring the fact that *B. melitensis* strains have become a severe public health issue in Jiangsu province and neighboring regions. Due to livestock trade and transfer, multiple *B. melitensis* lineages eventually trigger a local human brucellosis epidemic and spread. This investigation provides a vital cluse for devising and exerting a tailor control measure; however, further genomic epidemiological analysis on a regional and national scale will provide more detailed information to understand the transmission pattern of *B. melitensis* from north to south.

## Data Availability

The datasets generated during the current study are available in the National Microbiology Data Center repository (nmdc.cn), with accession number SUB1738305701652.
